# Effect of attachment styles on general psychological well-being and resilience as a mediating factor

**DOI:** 10.1192/j.eurpsy.2025.1472

**Published:** 2025-08-26

**Authors:** M. A. Elgendy, M. Hany, N. Wafa, M. Hesham, A. Elsayed, D. N. K. Boulos

**Affiliations:** 1 Newgiza University, Cairo, Egypt

## Abstract

**Introduction:**

Attachment styles, developed through early life interactions, influence how individuals perceive relationships and manage stress. Medical students often exhibit insecure attachment styles, which are linked to increased psychological distress.

**Objectives:**

The primary aim of this study is to explore the extent to which resilience influences the connection between attachment styles and general psychological well-being among medical students.

**Methods:**

This cross-sectional study was conducted at New Giza University in Cairo, Egypt, and involved 437 medical students. Participants completed self-report surveys measuring attachment styles, resilience, and psychological distress. The survey instruments included the Relationship Questionnaire (RQ) for attachment styles, the General Health Questionnaire (GHQ-12) for psychological distress (where a lower GHQ-12 score indicates less psychological distress), and the Connor–Davidson Resilience Scale (CD-RISC-10) for resilience. Participants were grouped into four attachment styles: secure (SA), fearful avoidant (FA), anxious preoccupied (AP), and dismissive avoidant (DA).

**Results:**

The sample’s mean age was 20.32 years (SD = 2.09). Females comprised 69.6% of the cohort, and single students made up 80.1% of the whole cohort. The distribution of attachment styles across females and males differed significantly (p < 0.0001, χ² = 25.7), with DA being more prevalent in males compared to females (28.6% and 13.2%, respectively). GHQ-12 median scores were similar between genders (p = 0.23), while CD-RISC-10 scores differed (p = 0.002), with males having a higher median (28).

Median scores of GHQ-12 and CD-RISC-10 differed significantly across groups of attachment styles (p < 0.0001 and p = 0.0002, respectively). AP and FA had the highest GHQ-12 medians (15), while AP had the lowest CD-RISC-10 median (25).

A negative correlation between CD-RISC-10 and GHQ-12 scores was significant in the whole cohort (p < 0.0001, r = -0.3227), with FA showing the strongest correlation (p < 0.0001, r = -0.3292, CI: -0.4714 to -0.1704) and other groups showing similar reuslts (figures 1, 2 and 3).

**Image:**

**Figure:**
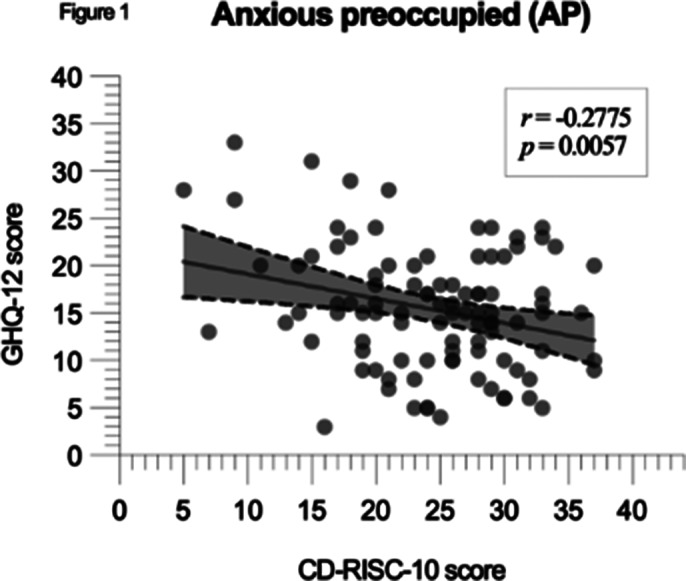

**Image 2:**

**Figure:**
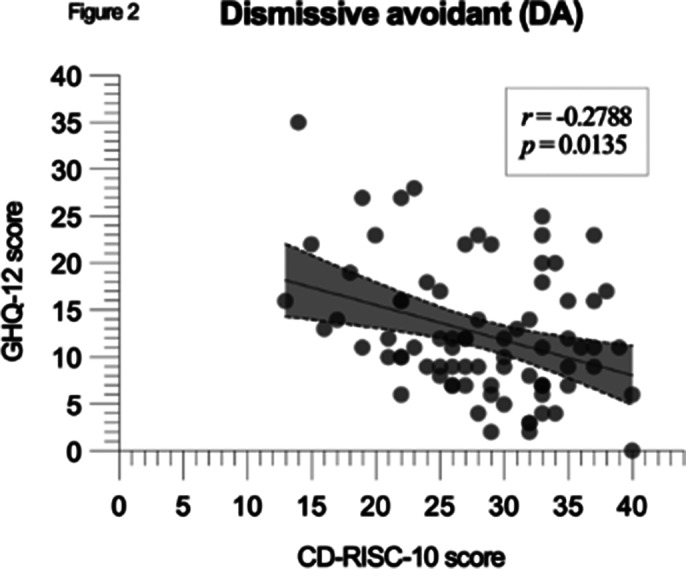

**Image 3:**

**Figure:**
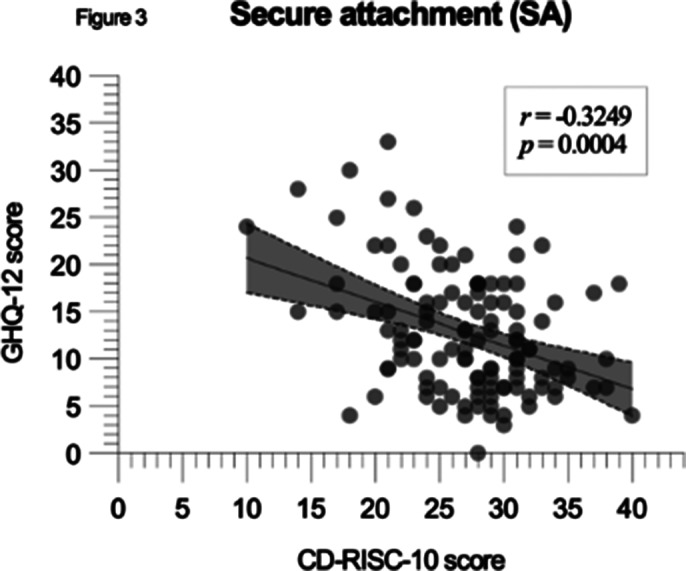

**Conclusions:**

The findings underscore the critical role of resilience in buffering against the psychological impacts of insecure attachment among medical students. Approaches targeting resilience enhancement could serve as a valuable intervention target to mitigate psychological distress and improve well-being in this high-risk attachment styles. Future research is recommended to develop and test resilience-focused interventions tailored to medical students with insecure attachment styles, which may help reduce burnout and promote mental health in medical training environments.

**Disclosure of Interest:**

None Declared

